# Supplementary material for: Therapeutic Effect of Rapamycin on TDP-43-Related Pathogenesis in Ischemic Stroke
Source: Int J Mol Sci. 2022 Dec 30;24(1):676. doi: 10.3390/ijms24010676 (PMC9820757; doi:10.3390/ijms24010676)
Supplement: Supplementary file 1 [file ijms-24-00676-s001.zip › ijms-2027637-supplementary.pdf]

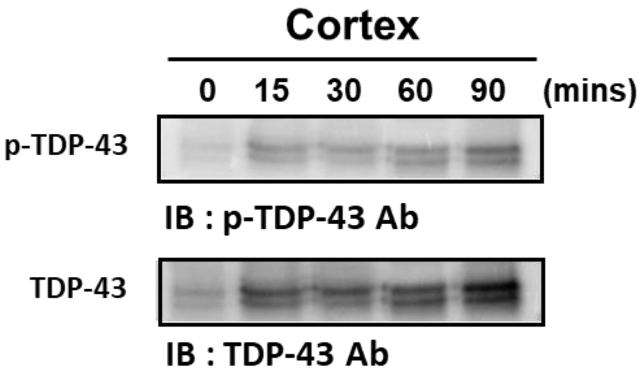

Figure S1. Pathological phosphorylated TDP-43 was specifically expressed in the right ischemic cortex region in a time-dependent manner. Transient focal cerebral ischemia was induced using the right MCA (middle cerebral artery) occlusion model in rats. After 15, 30, 60 and 90 min of ischemia, the brain tissues were dissected from these ischemic rats. The phosphorylated TDP-43 was significantly increased in the right ischemic cortex region in a time-dependent manner.
